# Slowly Digestible Carbohydrate Diet Ameliorates Hyperglycemia and Hyperlipidemia in High-Fat Diet/Streptozocin-Induced Diabetic Mice

**DOI:** 10.3389/fnut.2022.854725

**Published:** 2022-04-15

**Authors:** Yu-Zhong Chen, Jia Gu, Wei-Ting Chuang, Ya-Fang Du, Lin Zhang, Meng-Lan Lu, Jia-Ying Xu, Hao-Qiu Li, Yan Liu, Hao-Tian Feng, Yun-Hong Li, Li-Qiang Qin

**Affiliations:** ^1^State Key Laboratory of Radiation Medicine and Protection, School of Radiation Medicine and Protection, Soochow University, Suzhou, China; ^2^Inner Mongolia Dairy Technology Research Institute Co., Ltd., Hohhot, China; ^3^Department of Nutrition and Food Hygiene, School of Public Health, Soochow University, Suzhou, China

**Keywords:** slowly digestible carbohydrate, hyperglycemia, hyperlipidemia, high-fat diet, streptozocin, diabetes

## Abstract

**Objective:**

Given that the prevalence rate of type 2 diabetes mellitus (T2DM) continues to increase, it is important to find an effective method to prevent or treat this disease. Previous studies have shown that dietary intervention with a slowly digestible carbohydrate (SDC) diet can improve T2DM with almost no side effects. However, the underlying mechanisms of SDC protect against T2DM remains to be elucidated.

**Methods:**

The T2DM mice model was established with a high-fat diet and streptozocin injection. Then, SDC was administered for 6 weeks. Bodyweight, food intake, organ indices, fasting blood glucose (FBG), oral glucose tolerance test (OGTT), homeostasis model assessment for insulin resistance (HOMA-IR), and other biochemical parameters were measured. Histopathological and lipid accumulation analyses were performed, and the glucose metabolism-related gene expressions in the liver and skeletal muscle were determined. Lastly, colonic microbiota was also analyzed.

**Results:**

SDC intervention alleviated the weight loss in the pancreas, lowered blood glucose and glycosylated hemoglobin levels, and improved glucose tolerance and HOMA-IR. SDC intervention improved serum lipid profile, adipocytokines levels, and lowered the lipid accumulation in the liver, subcutaneous adipose tissue, and epididymal visceral adipose tissue. In addition, SDC intervention increased the expression levels of IRS-2 and GLUT-2 in liver tissues and elevated GLUT-4 expression levels in skeletal muscle tissues. Notably, SDC intervention decreased the Bacteroidetes/Firmicutes ratio, increased *Desulfovibrio* and *Lachnospiraceae* genus levels, and inhibited the relative abundance of potentially pathogenic bacteria.

**Conclusions:**

SDC intervention can improve hyperglycemia and hyperlipidemia status in diabetic mice, suggesting that this intervention might be beneficial for T2DM.

## Introduction

Diabetes mellitus (DM) is an important global public health problem. Currently, over 415 million people live with DM, and the number is estimated to rise to 642 million by 2040 (1). Around 90% of DM patients have type 2 diabetes mellitus (T2DM) (2). T2DM is characterized by hyperglycemia and is accompanied by the dysfunction of carbohydrate, fat and protein metabolism (3). Hyperglycemia in T2DM patients is caused by impaired insulin secretion and insulin resistance (IR) (4). Since glucose-lowering medications may have side effects, T2DM patients often prefer complementary and alternative medications considering their minimal or negligible side effects. The American Diabetes Association recommended dietary intervention as one of the non-pharmacological approaches to prevent and manage T2DM (5).

Currently, it is recommended that 45–60% energy of the daily total calorie intake for T2DM patients should come from carbohydrates (6). The American Diabetes Association suggested that the carbohydrate quality, rather than their quantity, is key in the care of T2DM patients (7). Foods that provide high-quality carbohydrates with a slow energy supply, low glycemic index, dense nutrition, and desirable sensory properties are preferred for diabetic patients (8). Slowly digestible carbohydrates (SDCs) are digested gradually, allowing a steady release of glucose from the small intestine into the bloodstream, thereby blunting postprandial glucose surges and lowering insulin requirement (9). Thus, foods with a high SDCs content might possess a protective effect against T2DM. Previous studies revealed that SDCs benefit glycemic control in T2DM patients (8–11), although the mechanisms for this benefit remain unclear.

Streptozotocin (STZ) is an alkylating agent toxic to insulin-producing pancreatic beta cells, and its injection into mice or rats provokes insulin deficiency and hyperglycemia (12). The combination of high-fat diet (HFD) and low-dose STZ is often used to establish a diabetic animal model that mimics human T2DM (IR and initial beta cell dysfunction) (13). In the present study, high-fat diet/streptozotocin (HFD/STZ)-induced diabetic mice were intervened with SDC to investigate: (1) the potential protective effect of SDC against hyperglycemia and hyperlipidemia; (2) the effect of SDC on glucose uptake and utilization in the liver and skeletal muscle tissues; (3) the effect of SDC on colonic flora composition.

## Materials and Methods

### Animals

Male C57BL/6J mice (6–8 weeks old) were purchased from Jihui Laboratory Animal Co. Ltd. (Shanghai, China) and housed in the Laboratory Animal Center of Soochow University. The mice had free access to food and water *ad libitum* and were maintained in a specific pathogen-free (SPF) room under a 12 h light-dark cycle, temperature of 22–25°C, and relative humidity of 60 ± 5%. The study protocol has been reviewed and approved by the Animal Ethical Committee of Soochow University, China (Certificate No. XCYK[Su]2002-0008).

### Induction of a T2DM Model in Mice

**Figure 1:**
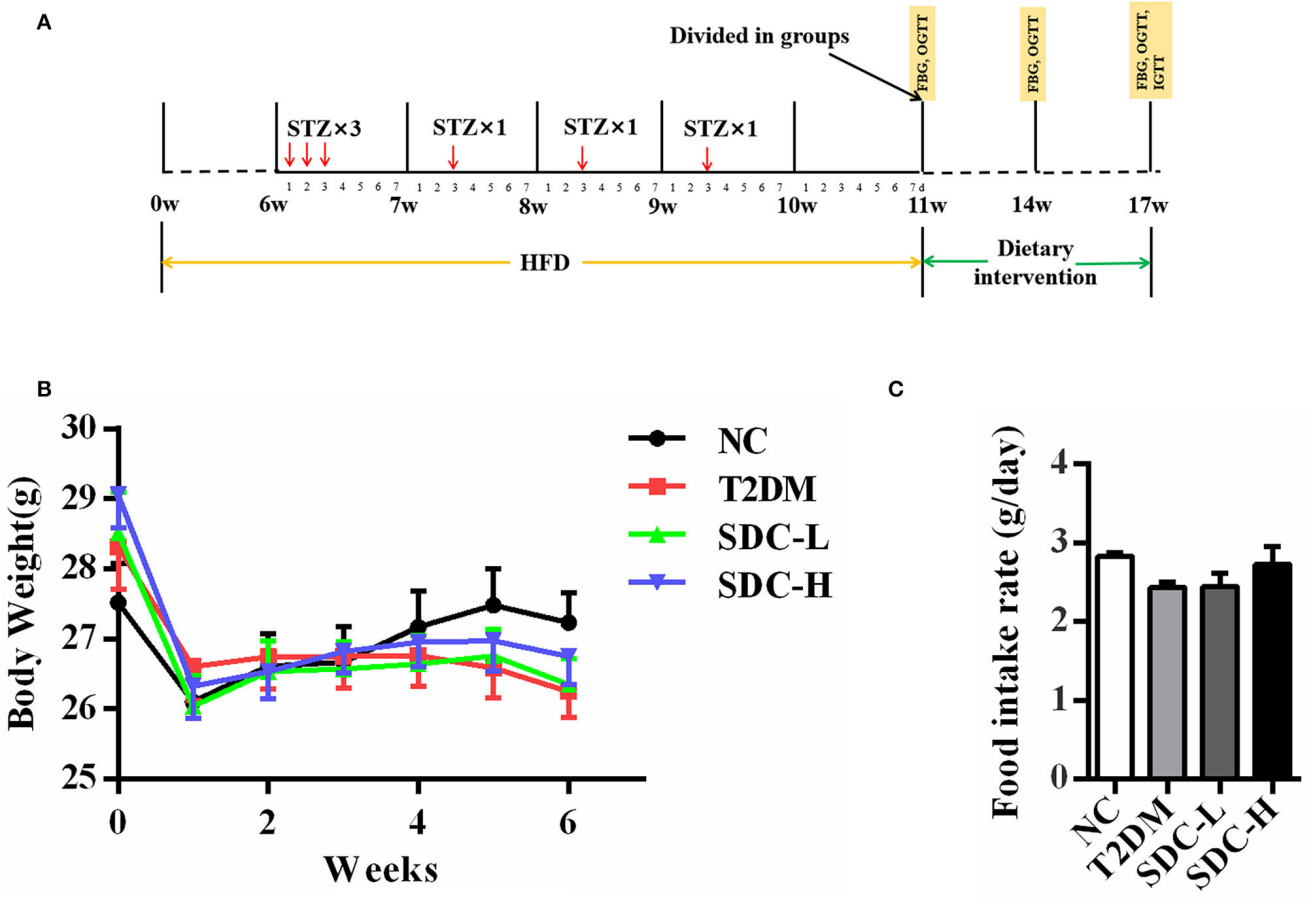
**(A)** Schematic representation of experimental procedure; **(B)** Body weight of the mice during the intervention period, *n* = 11; **(C)** Food intake of the mice during the intervention period, *n* = 11.

After acclimating for 1 week, 11 mice were fed with a normal diet [American Institute of Nutrition, 1993, Maintenance (AIN-93M)] (normal control, NC), while the others (45 mice) were given a HFD (60% energy from fat, Dyets, Inc., Bethlehem, PA) for 11 weeks. The HFD/STZ induced T2DM model was established according to a previous study with modifications (14). After being fed with a HFD for 6 weeks, the mice fed with a HFD were given an intraperitoneal injection of 40 mg/kg STZ (dissolved in 0.01 M citrate buffer with pH 4.5 before use) for 6 times. Meanwhile, the mice in the NC group were injected with citrate buffer (0.01 M; pH 4.2–4.5). One week after the last STZ injection, the fasting blood glucose (FBG) levels of the mice were measured. The mice with FBG levels within 11.1–16.7 mmol/L after STZ injection were considered T2DM and were randomly divided into 3 groups of 11 mice per group (15, 16). The detailed experimental procedure is outlined in Figure 1A.

### Experimental Design

After the T2DM mice model was established, the mice were allocated into 4 groups (*n* = 11) as follows: non-diabetic mice fed with an AIN-93M diet (NC group); HFD/STZ-induced diabetic mice fed with an AIN-93M diet (T2DM Group); T2DM mice administered a low dose of SDC (50% carbohydrate substituted, contained Roquette cassava dextrin, 79.45 g/kg; Tate & Lyle maltodextrin (Dextrose equivalent value = 5), 206.33 g/kg; Hi-maize resistant starch, 9.01 g/kg; Nubana green banana powder, 5.45 g/kg; trehalose, 37.84 g/kg; and palatinose, 34.23 g/kg) for 6 weeks (T2DM+SDC-L Group); T2DM mice administered a high dose of SDC (100% carbohydrate substituted, contained double doses of cassava dextrin, DE = 5 maltodextrin, resistant starch, green banana powder, trehalose, and palatinose compared to the SDC-L group) for 6 weeks (T2DM+SDC-H Group). The diet compositions of AIN-93M, HFD, SDC-L and SDC-H were listed in Table 1.

**Table 1:** Ingredients and chemical composition of experimental diets.

**Ingredient g/kg diet**	**AIN-93M diet**	**HFD**	**SDC-L diet**	**SDC-H diet**	**Caloric value (kcal/g)**
Caisein	140	258.45	139.72	139.43	4
L-Cystine	1.8	3.88	1.8	1.8	4
Sucrose	100	88.91	50	0	4
Cornstarch	465.692	0	232.85	0	4
Dyetrose	155	161.53	77.5	0	4
Soybean Oil	40	32.31	39.99	39.98	9
Lard	0	316.6	0	0	9
Cellulose	50	64.61	39.32	28.64	0
Mineral Mix#210050	35	0	34.002	33.042	0
Mineral Mix#210088	0	12.92	0	0	1.6
Calcium carbonate	0	7.11	0	0	0
Dicalcium phosphate	0	16.8	0	0	0
Potassium citrate H_2_O	0	21.32	0	0	0
Vitamin Mix#310025	10	0	10	10	4
Vitamin Mix#300050	0	12.92	0	0	3.9
Choline bitartrate	2.5	2.58	2.5	2.5	0
Blue dye	0	0.06	0	0	0
t-Butylhydroquinone	0.008	0	0.008	0.008	0
Roquette cassava dextrin	0	0	79.45	158.89	3.8
Tate & Lyle maltodextrin	0	0	206.33	412.65	4
Hi-maize resistant starch	0	0	9.01	18.02	1.6
Nubana green banana powder	0	0	5.45	10.9	2.6
Trehalose	0	0	37.84	75.67	4
Palatinose	0	0	34.23	68.47	4
Total mass (g)	1,000.00	1,000.00	1,000.00	1,000.00	/
Total caloric value (kcal)	3.85 × 10^3^	5.26 × 10^3^	3.85 × 10^3^	3.85 × 10^3^	/

During the dietary intervention period, the bodyweight of each mouse and the food intake of each cage were measured weekly. After 6 weeks of intervention, the mice were fasted for 12 h, anesthetized, and then blood samples were collected from the orbital venous plexus. The blood samples were allowed to clot at room temperature for 30 min and centrifuged at 3,000 × *g* for 10 min, and then the plasma was collected. After the mice were sacrificed, the liver, kidney, spleen, pancreas, subcutaneous fat tissue, epididymal fat tissue, and perirenal fat tissue were weighed and collected for further analysis. The organ indices were expressed relative to body weight (g of organ/kg of body weight) (17). Colonic contents were collected in sterile tubes and stored at −80°C until analysis.

### Measurement of Fasting Blood Glucose Levels

The blood samples were collected from the tail vein to analyze FBG levels with a blood glucose meter (F. Hoffmann-La Roche Ltd., Basel, Switzerland) according to a previous study (18). FBG levels were monitored 3 times during the dietary intervention period at the 0th, 3rd, and 6th week, corresponding to the 11th, 14th, and 17th week during the whole experiment.

### Oral Glucose Tolerance Test

The OGTT test was performed at the 1st, 3rd, and 6th weeks after the initiation of dietary intervention according to a previous study (18). The mice in all groups were fasted overnight and then administered glucose orally (2 g/kg of body weight). The blood glucose levels were measured at 0, 30, 60, and 120 min after glucose administration, and the glucose area under the curve (AUC) was calculated by the trapezoidal rule.

### Biochemical Parameters Determination

The liver glycogen content and serum concentrations of lipid parameters, including total triglycerides (TG), total cholesterol (TC), low-density lipoprotein cholesterol (LDL-C), and high-density lipoprotein cholesterol (HDL-C), were determined according to the manufacturer's instructions (Nanjing Jiancheng Bioengineering Institute, Nanjing, China). The levels of resistin (RES), adiponectin (ADPN), leptin (LEP), glycated hemoglobin (GHb), and C-peptide in serum were measured using enzyme-linked immunosorbent assay (ELISA) kits (Wuhan ColorfulGene Biological Technology Co., LTD). The fasting serum insulin (FINS) was measured using a Mercodia ELISA kit (Mercodia company, Uppsala, Sweden), and glucagon was measured using an Elabscience ELISA kit (Elabscience Biotechnology Co., Ltd, Wuhan, China). The homeostasis model assessment of insulin resistance (HOMA-IR) and quantitative insulin sensitivity check index (QUICKI) were calculated according to the method described earlier by using the following formulas (19):


(1) $$\begin{array}{r} HOMA-IR=FBG\left(mmol/L\right)\;\times \;FINS\left(\mu U/mL\right)/22.5\; \end{array}$$

(2) $$\begin{array}{r} QUICKI=1/\left\{log\left(FBG\left[mg/dL\right]\right)+log\left(FINS\left[\mu U/mL\right]\right)\right\}\; \end{array}$$

### Histopathological Observation

The excised fragments of liver tissue, subcutaneous adipose tissue, and epididymal visceral adipose tissue were fixed in 10% neutral buffered formalin solution (12). The specimens were embedded in paraffin, cut in 4 μm thickness sections, and stained with hematoxylin and eosin (H&E). Then, the sections were observed under the Leica DMi8 light microscope (Leica Corp., Wetzlar, Germany). The average size of fat vacuole was calculated by Image J software (Version 1.8).

### Lipid Accumulation Analysis

The lipid accumulation in the sections of liver tissue, subcutaneous adipose tissue, and epididymal visceral adipose tissue was stained with oil red O according to a previous study with modification (20). The samples were fixed in a 10% neutral buffered formalin solution and stained with oil red O for 8 min. Then, the slides were differentiated in 60% isopropanol and rinsed with distilled water. Lastly, the samples were counterstained with hematoxylin at room temperature for 5 min, observed using a light microscope (Leica Corp., Wetzlar, Germany), and analyzed with Image J software (Version 1.8).

### Immunohistochemistry Analysis

The immunohistochemistry analysis was performed as described by a previous study with slight modification (21). The liver tissue and epididymal visceral adipose tissue sections of mice were deparaffinized in xylene, rehydration in graded alcohol, and antigen retrieval in citrate buffer (pH 6.0) at 100°C. The liver tissue slides were incubated in anti-IRS-2 antibody (Abcam, Cambridge, England, 1:500) and GLUT-2 primary antibody (Bioss Biotechnology Co., LTD, Beijing, China, 1:500) overnight. Then, the slides were incubated with a secondary antibody (goat antirabbit), stained with diaminobenzidine (DAB), and counterstained with hematoxylin. The stained sections were observed using a light microscope (Leica Corp., Wetzlar, Germany) and analyzed with the Image J software (Version 1.8).

### Quantitative Reverse Transcription Polymerase Chain Reaction Analysis

The RT-qPCR analysis was performed according to a previous study (22). The skeletal muscle tissues were mixed with TRIzol reagent (Tiangen Biotech Co., Ltd., Beijing, China) to extract the total RNA. The total RNA was quantified with the Nanodrop 2000 spectrophotometer (Thermo Fisher Scientific Inc., Waltham, MA, USA) and reverse transcribed to cDNA with a High-Capacity cDNA Reverse Transcription Kit (Thermo Fisher Scientific Inc. Waltham, MA, USA). The RT-qPCR was conducted with a ChamQ™ Universal SYBR green PCR master mix (Vazyme Biotech Co., Ltd, Nanjing, China) using the QuantStudio™ 6 Flex system (Thermo Fisher Scientific Inc., Waltham, MA, USA). The relative expression ratios of IRS-1 and GLUT-4 genes were normalized to the corresponding expression of GAPDH by using the 2^−ΔΔCT^ method. The sets of primers used for RT-qPCR were listed as follows.

GAPDH F: ACCCAGAAGACTGTGGATGG; GAPDH R: TCTAGACGGCAGGTCAGGTC; IRS-1 F: CGATGGCTTCTCAGACGTG; IRS-1 R: CAGCCCGCTTGTTGATGTTG; GLUT-4 F: GGTTGGTGCCTATGTATGT; GLUT-4 R: CGGATGATGTAGAGGTATCG.

### Western Blot Analysis

The western blot analysis was performed according to a previous study (22). The skeletal muscle tissue protein was extracted with RIPA lysis buffer (Beyotime Biotechnology, Shanghai, China) containing PMSF (4°C). The total protein concentration was determined using a BCA protein quantification kit (Beyotime Biotechnology, Shanghai, China). The proteins were loaded onto 10% sodium dodecyl sulfate-polyacrylamide gel for electrophoresis and then transferred to polyvinylidene fluoride membrane (Millipore, Billerica, MA, USA). The membranes were blocked in 5% skimmed milk solution for 1 h and incubated using primary antibodies: anti-IRS-1 (1:100), anti-GLUT-4 (1:200), and anti-β-actin (1:1,000) overnight at 4°C. After the incubation, the membranes were rinsed three times in Tris-buffered saline Tween-20 (TBST), and incubated with the secondary antibodies (1:1,000) for 1 h. After washing with three TBST washes, the bands were visualized under a G:BOX Chemi XL1.4 Gel imaging and analysis system (Syngene Corp., Cambridge, United Kingdom). The band images were analyzed using the ImageJ software (Version 1.8), and the relative expression levels of the target proteins were normalized to the expression of β-actin.

### Gut Microbiota Analysis

Genomic DNA was directly extracted from colonic contents of mice with an E.Z.N.A. ^®^Stool DNA Kit (D4015, Omega, Inc., USA), and DNA integrity was assessed by 1% agarose gel electrophoresis. The primers 341F/805R (341F:5′-CCTACGGGNGGCWGCAG-3′;805R:5′-GACTACHVGGGTATCTAATCC-3′) were used for amplification of the V3-V4 region of the bacteria 16S rRNA gene (23). Then, the resulting PCR products were extracted with 2% agarose gels and purified using AMPure XT beads (Beckman Coulter Genomics, Danvers, MA, USA). The purified amplicons were quantified with the Qubit assay kit (Invitrogen, USA). The size and quantity of the amplicon library were assessed on the Agilent 2100 Bioanalyzer (Agilent, USA) and with the Library Quantification Kit for Illumina (Kapa Biosciences, Woburn, MA, USA), respectively. The libraries were sequenced on the Illumina NovaSeq PE250 platform. After filtering the data, the sequencing data analysis, including alpha-diversity and beta-diversity, was calculated with the QIIME2. The graphs of beta diversity were drawn with the R package.

### Statistical Analysis

Data were presented as mean ± standard error of the mean (SEM). Differences between groups were assessed using the ANOVA followed by LSD *post-hoc* test with SPSS version 24.0 software. All *P*-values were two-sided, and the significance level was set at <0.05.

## Results

### Effect of SDC on Body Weight, Food Intake, and Organ Indices

During the dietary intervention period, there were no considerable differences in body weight and food intake across the groups (Figures 1B,C). Moreover, the differences in indices of the spleen, subcutaneous adipose tissue, and visceral adipose tissue were not significant (Table 2). Increased liver index (*P* < 0.001) and reduced pancreas index (*P* < 0.01) were observed in the T2DM group when compared with the NC group. This result suggested that HFD/STZ induced diabetic hepatomegaly and pancreas atrophy in T2DM mice, which was consistent with a previous study (24). After SDC intervention, the liver index was decreased (*P* < 0.001), and the pancreas index was slight improved in the SDC-H group. This demonstrated that the liver enlargement and pancreas atrophy had been improved after SDC-H diet intervention. However, the kidney index was significantly lower in the SDC-H group, suggesting that a high dose of SDC might impair kidney function.

**Table 2:** Effect of SDC on organ indices.

**Organ indices (g/kg of body weight)**	**NC**	**T2DM**	**SDC-L**	**SDC-H**
Liver	53.58 ± 1.22	59.09 ± 1.08^***^	59.49 ± 1.06^***^	51.73 ± 0.91^###^
Kidney	13.77 ± 0.27	13.18 ± 0.2	13.22 ± 0.23	12.44 ± 0.27^***^^#^
Spleen	3.1 ± 0.27	2.97 ± 0.14	3.09 ± 0.33	3.16 ± 0.19
Pancreas	5.59 ± 0.58	2.9 ± 0.64^**^	4.01 ± 0.53^*^	4.02 ± 0.44
Subcutaneous fat	13.97 ± 0.94	13.31 ± 0.99	12.56 ± 0.8	13.28 ± 0.74
Visceral fat	23.82 ± 1.66	24.96 ± 1.27	21.41 ± 1.33	26.16 ± 0.89

*n = 11*.

* *P < 0.05 vs. NC*.

** *P < 0.01 vs. NC*.

*** *P < 0.001 vs. NC*.

# *P < 0.05 vs. T2DM*.

### *P < 0.001 vs. T2DM*.

### Effect of SDC on FBG, OGTT, GHb, and Liver Glycogen

**Figure 2:**
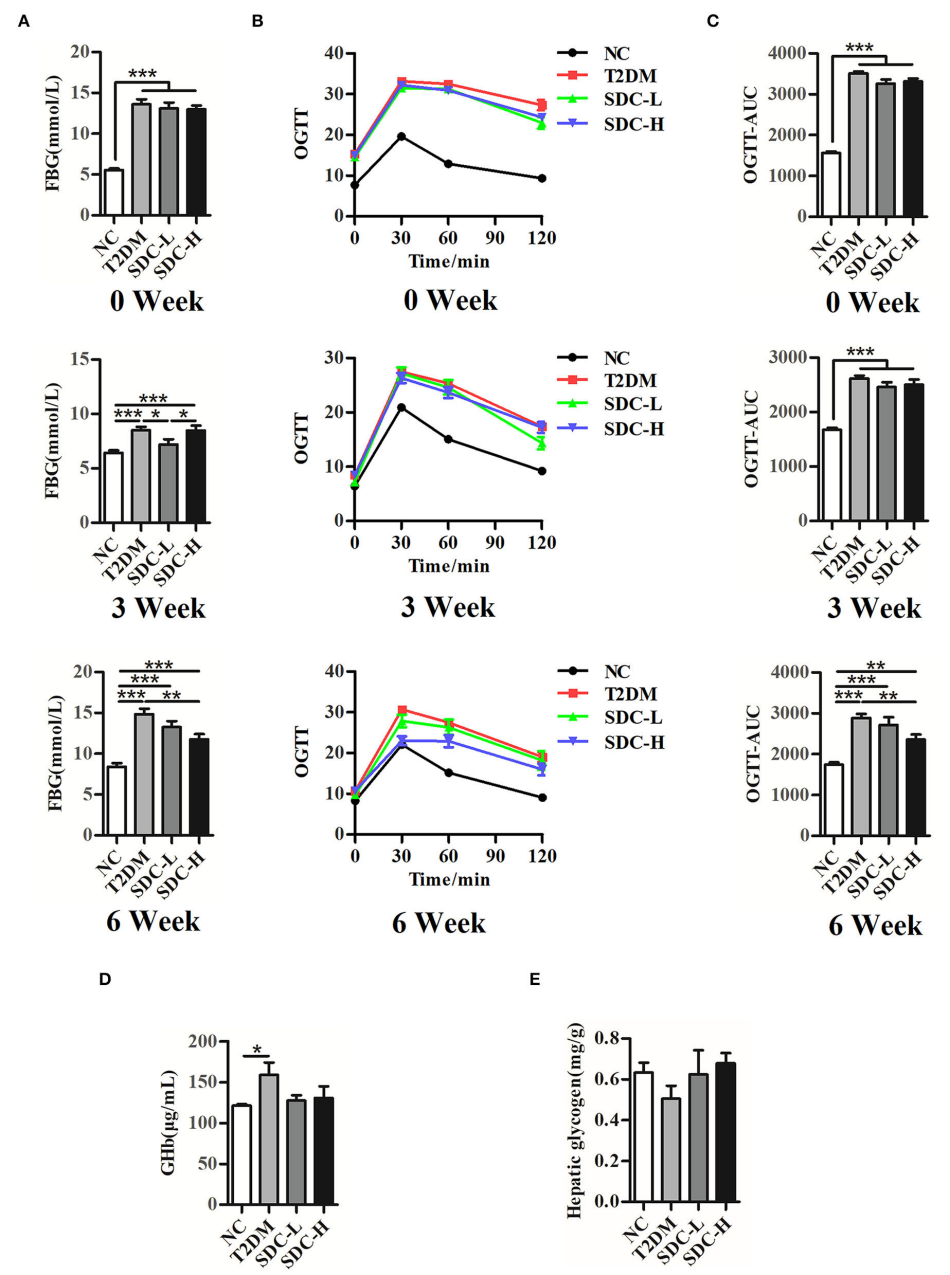
FBG **(A)**, OGTT **(B)**, and **(C)** OGTT-AUC of the mice at 0th, 3rd, 6th week after the intervention begin, *n* = 11; **(D)** GHb levels of the mice, *n* = 8; **(E)** Hepatic glycogen contents of the mice, *n* = 5. *P < 0.05, **P < 0.01, ***P < 0.001.

As shown in Figure 2, the FBG (*P* < 0.001) and AUC_0−120minGlu_ (*P* < 0.001) levels were significantly increased in the T2DM group at the 0th, 3rd, and 6th week after the dietary intervention start. The GHb levels in the T2DM group were significantly higher than in the NC group (*P* < 0.05), suggesting that hyperglycemia existed in the mice. In contrast, the mice in the SDC-H group showed reductions in FBG (*P* < 0.01), AUC_0−120minGlu_ (*P* < 0.01), and GHb levels (*P* > 0.05). Thus, SDC intervention improved the hyperglycemic status of mice. The liver is an important organ for maintaining glucose homeostasis. The liver glycogen storage decreased in the T2DM mice, indicating that hepatic glucose output was increased. Subsequently, SDC treatment enhanced the liver glycogen levels (*P* > 0.05), suggesting that SDC can improve the glucose homeostasis of diabetic mice.

### Effect of SDC on Glucagon, C-Peptide, Insulin, HOMA-IR, and QUICKI

T2DM is often accompanied by hyperglucagonemia, a contributing factor to diabetic hyperglycemia (25). Indeed, the serum glucagon level of T2DM mice was increased (*P* < 0.001), and SDC intervention decreased the glucagon level to some extent (*P* > 0.05). C-peptide is produced in equal amounts to endogenous insulin and is useful for assessing pancreatic beta-cell function. C-peptide levels decline with the duration of diabetes (26). Compared to the NC group, the level of C-peptide was significantly decreased in the T2DM group (*P* < 0.05). There were no differences in C-peptide levels between the SDC intervention groups and the T2DM group. The insulin level (*P* < 0.05) and HOMA-IR (*P* < 0.001) were increased, and the QUICKI (*P* < 0.001) was decreased in the T2DM group. After SDC intervention, these changes were reversed both in SDC-L and SDC-H groups (Table 3). These results indicated that SDC intervention could improve the IR status in T2DM mice.

**Table 3:** Effect of SDC on glucagon, C-peptide, insulin, HOMA-IR, and QUICKI.

**Parameters**	**NC**	**T2DM**	**SDC-L**	**SDC-H**
Serum glucagon (pg/mL)	358.44 ± 11.61	478.49 ± 21.93^***^	454.44 ± 14.5^**^	423.62 ± 31.09^*^
Serum C-peptide (pg/mL)	908.92 ± 20.18	832.85 ± 20.59^**^	859.9 ± 11.39	841.14 ± 15.62^*^
Serum insulin (μg/L)	0.23 ± 0.02	0.33 ± 0.04^*^	0.23 ± 0.04^#^	0.22 ± 0.03^#^
HOMA-IR	2.09 ± 0.21	5.27 ± 0.69^***^	3.14 ± 0.53^##^	2.74 ± 0.37^###^
QUICKI	0.61 ± 0.02	0.49 ± 0.01^***^	0.56 ± 0.03^#^	0.58 ± 0.02^##^

*n = 8*.

* *P < 0.05 vs. NC*.

** *P < 0.01 vs. NC*.

*** *P < 0.001 vs. NC*.

# *P < 0.05 vs. T2DM*.

## *P < 0.01 vs. T2DM*.

### *P < 0.001 vs. T2DM*.

### Effect of SDC on Serum Lipid Parameters and Adipokines

The serum lipid parameters levels were reported in Table 4. Compared to the NC group, the TC (*P* < 0.01) and LDL-C (*P* < 0.001) levels were increased, and HDL-C levels were decreased (*P* < 0.01) in the T2DM group, suggesting that abnormal lipid metabolism existed in the T2DM mice. After the administration of SDC, these lipid parameters showed an opposite trend in the SDC-H group. Specifically, the levels of TC and LDL-C group were decreased in the SDC-H group (*P* < 0.001).

**Table 4:** Effect of SDC on serum lipid parameters and adipokines.

**Parameters**	**NC**	**T2DM**	**SDC-L**	**SDC-H**
TG (mmol/L)	0.73 ± 0.02	0.71 ± 0.05	0.71 ± 0.04	0.79 ± 0.05
TC (mmol/L)	3.8 ± 0.16	4.44 ± 0.15^**^	4.76 ± 0.06^***^	3.75 ± 0.09^###^
LDL-C (mmol/L)	5.61 ± 0.19	6.94 ± 0.26^***^	7.72 ± 0.09^***^^#^	5.09 ± 0.28^###^
HDL-C (mmol/L)	1.3 ± 0.1	0.87 ± 0.12^**^	0.42 ± 0.06^***^^##^	1 ± 0.1^*^
RES (×10^3^ pg/mL)	2.63 ± 0.06	2.92 ± 0.07^**^	2.68 ± 0.05^##^	2.61 ± 0.05^###^
ADPN (pg/mL)	305.21 ± 6.03	282.09 ± 10.12^*^	304.34 ± 4.84^#^	293.78 ± 4.88
LEP (×10^3^ pg/mL)	1.7 ± 0.05	1.28 ± 0.08^***^	1.64 ± 0.06^###^	1.64 ± 0.04^###^

*TG, TC, LDL-C, and HDL-C, n = 6. RES, ADPN and LEP, n = 8*.

* *P < 0.05 vs. NC*.

** *P < 0.01 vs. NC.*

*** *P < 0.001 vs. NC*.

# *P < 0.05 vs. T2DM*.

## *P < 0.01 vs. T2DM*.

### *P < 0.001 vs. T2DM*.

The serum metabolic hormones (RES, ADPN, and LEP) levels were also presented in Table 4. Increased RES levels (*P* < 0.01) and decreased ADPN (*P* < 0.05) and LEP (*P* < 0.001) levels were observed in the T2DM when compared to the NC group. The levels of RES and LEP were improved in both SDC-L and SDC-H groups. In the SDC-L group, the level of ADPN was also significantly restored.

### Effect of SDC on Liver, Subcutaneous Adipose, and Epididymal Visceral Adipose Tissues Histopathology

**Figure 3:**
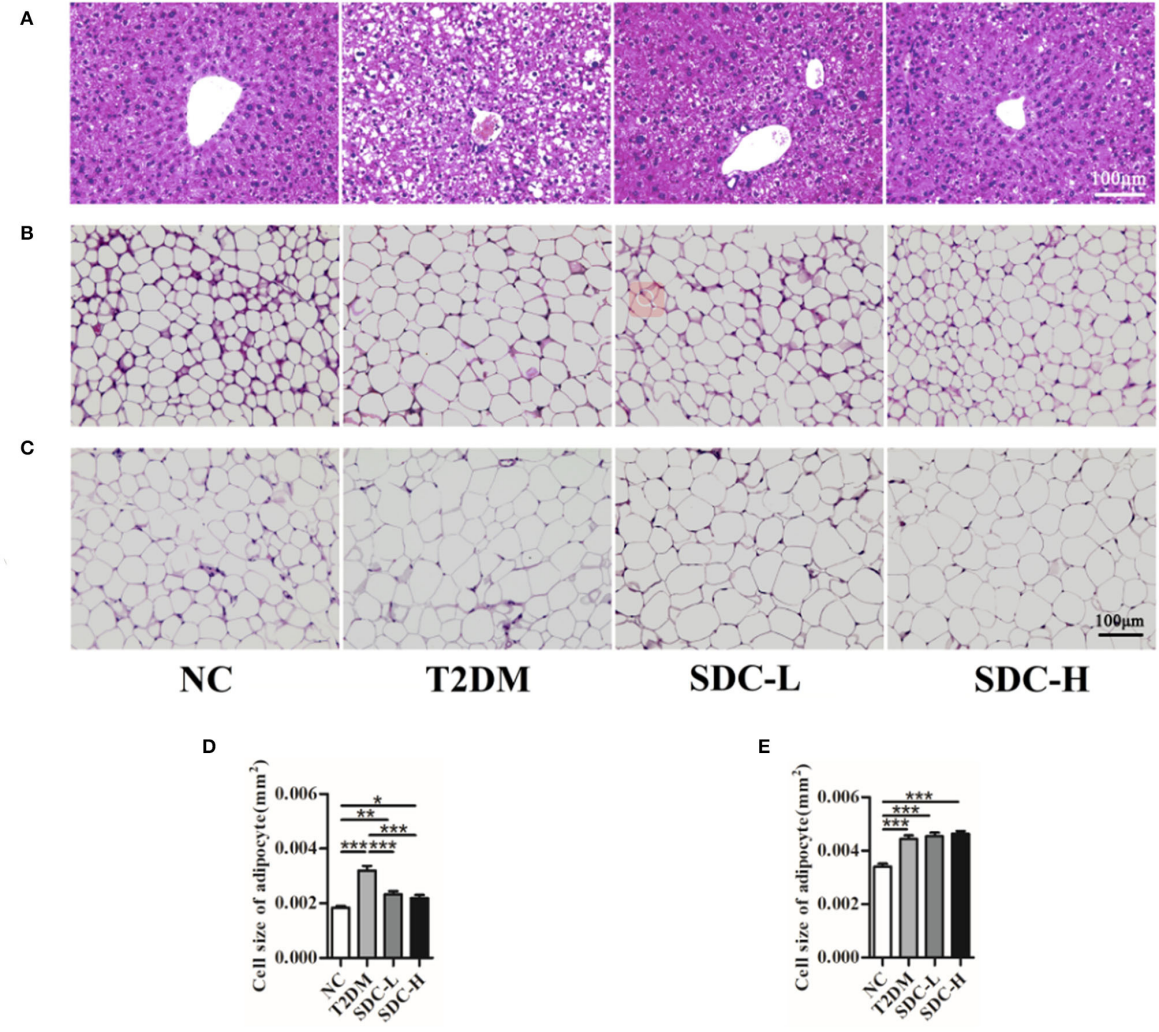
Liver **(A)**, subcutaneous adipose **(B)**, and epididymal visceral adipose **(C)** histopathology; Cell size of subcutaneous adipose tissue **(D)** and epididymal visceral adipose tissue **(E)**. *n* = 10, *P < 0.05, **P < 0.01, ***P < 0.001.

As shown in Figure 3A, the hepatic architecture of the mice in the NC group was normal, whereas the hepatocytes of the mice in the T2DM group showed irregular arrangement, steatosis and vacuolar degeneration. These pathological changes were mild in mice intervened with SDC, which indicated restoration of the hepatic structure. The light photomicrographs of subcutaneous adipose and epididymal visceral adipose tissues sections were displayed in Figures 3B,C. The fat cell size was larger in the T2DM group than in the NC group (Figures 3D,E). SDC intervention significantly decreased the symptom of subcutaneous adipose tissue (Figure 3D) but did not affect the epididymal visceral adipose tissue (Figure 3E).

### Effect of SDC on Lipid Accumulation in Liver, Subcutaneous Adipose, and Epididymal Visceral Adipose Tissues

**Figure 4:**
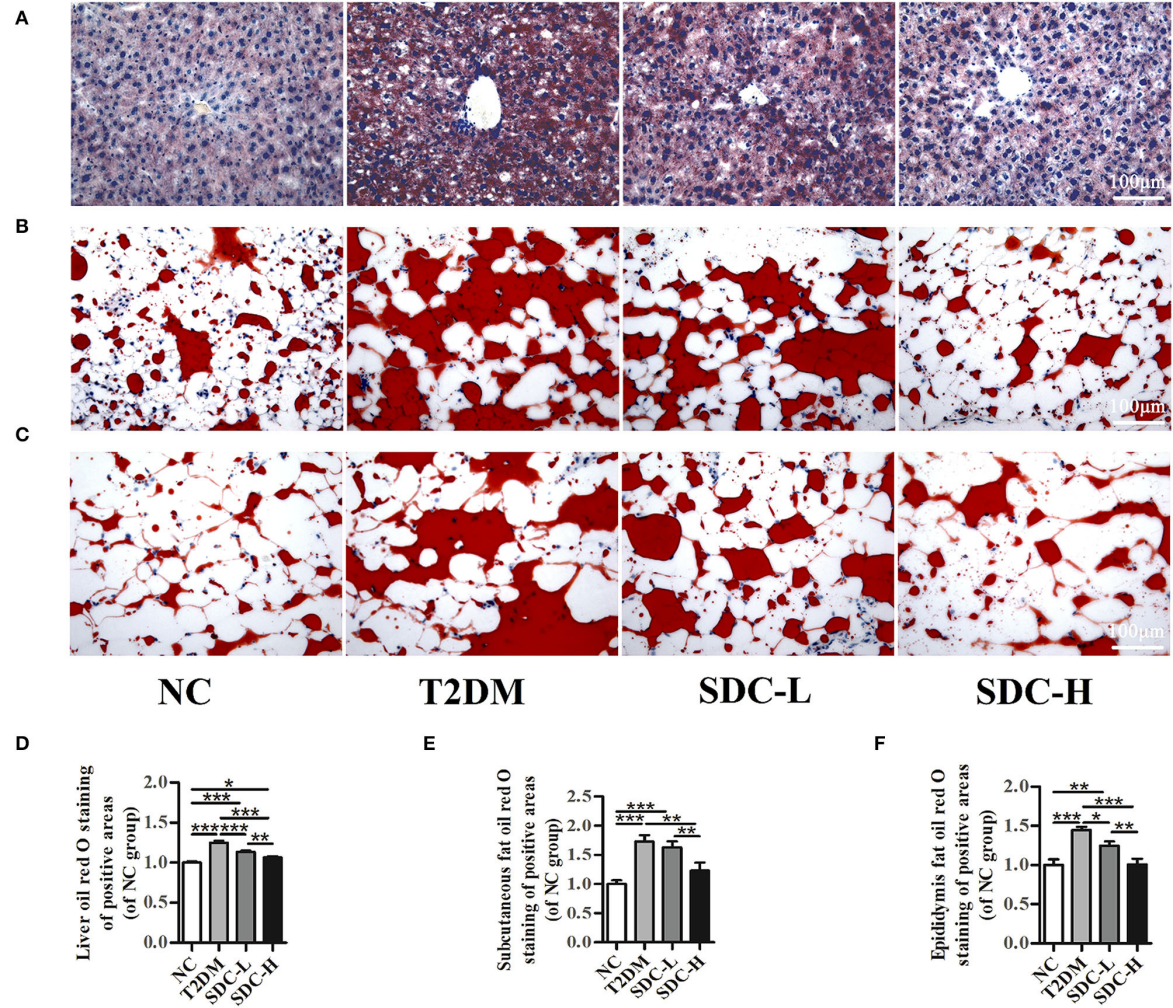
Lipid accumulation in liver **(A)**, subcutaneous adipose **(B)**, and epididymal visceral adipose **(C)**; Relative liver **(D)**, subcutaneous adipose **(E)**, and epididymal visceral adipose **(F)** oil red O red staining of positive areas. *n* = 5, ^*^P < 0.05, ^**^P < 0.01, ^***^P < 0.001.

Oil red O staining was performed to assess the effect of SDC on lipid accumulation in T2DM mice. The light microscopy observation revealed that liver cells in the NC group were arranged neatly and displayed almost no lipid droplet vacuoles. In contrast, large amounts of lipid droplets were distributed in the hepatocytes of the T2DM group. The high-intensity areas of lipid droplets were smaller in the SDC-L and SDC-H groups than in the T2DM group (Figure 4A), revealing that the hepatic steatosis was improved after SDC intervention. Similarly, lipid deposits were higher in subcutaneous adipose (Figure 4B) and epididymal visceral adipose (Figure 4C) tissues in the T2DM group than in the NC group; only smaller lipid droplets were displayed in the SDC intervention groups, especially in the SDC-H group. The relative liver, subcutaneous adipose, and epididymal visceral adipose oil red O red staining of positive areas were shown in Figures 4D–F, respectively.

### Effect of SDC on the Expression of Genes Related to Glucose Metabolism in Liver and Skeletal Muscle Tissues

**Figure 5:**
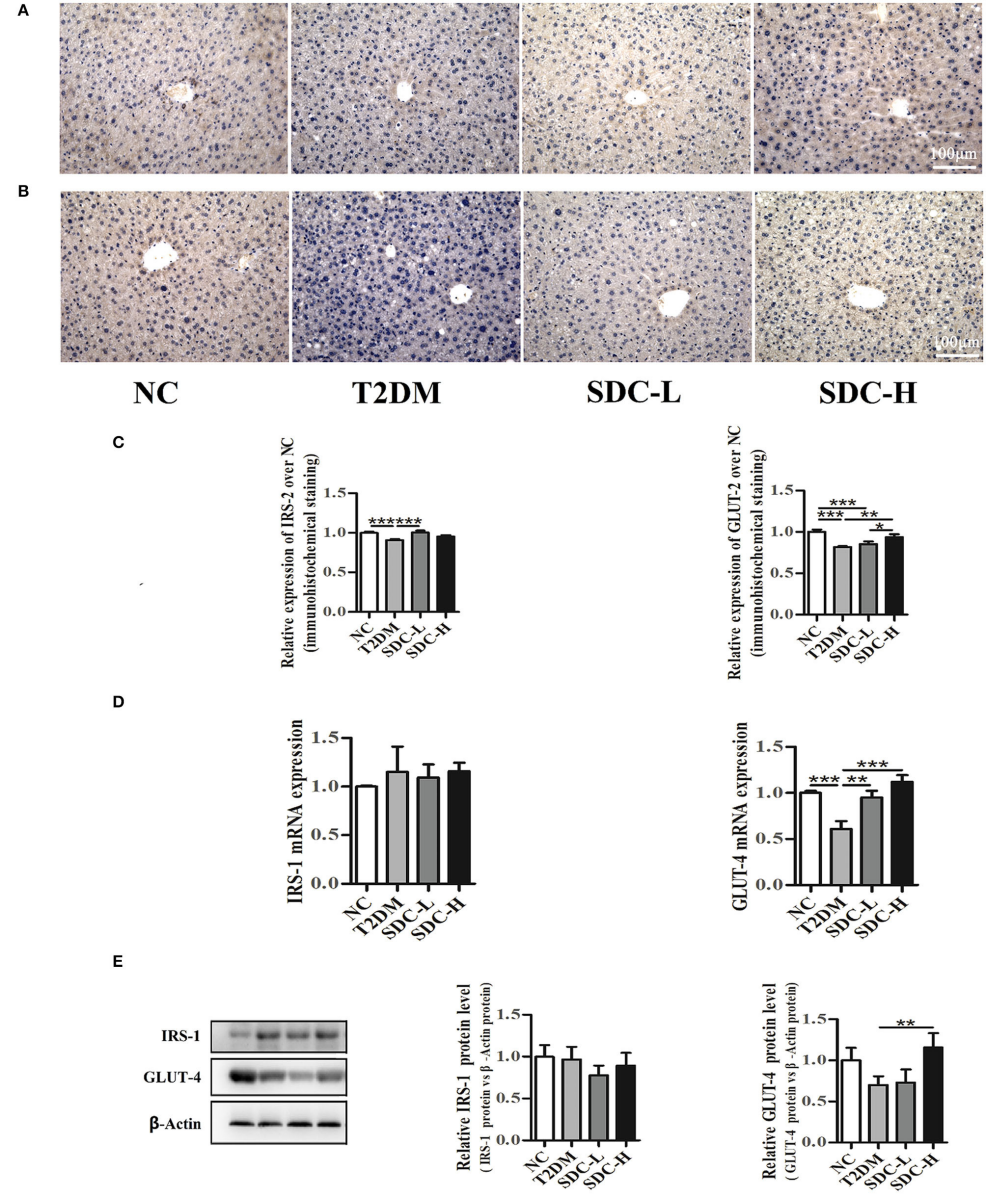
Immunohistochemistry analysis of IRS-2 **(A)** and GLUT-2 **(B)** in liver tissues, *n* = 5; Relative expression of IRS-2 and GLUT-2 in liver tissues, *n* = 5 **(C)**; mRNA levels of IRS-1 and GLUT-4 in skeletal muscle tissues, *n* = 8 **(D)**; protein levels of IRS-1 and GLUT-4 in skeletal muscle tissues, *n* = 6 **(E)**. *P < 0.05, **P < 0.01, ***P < 0.001.

Insulin receptor substrate (IRS) proteins played a crucial role in mediating insulin/IGF-1 signals. The downregulation of IRS proteins is highly related to IR status in rodents and humans (27). In the present study, immunohistochemistry analysis (Figures 5A,C) of the liver revealed that IRS-2 expression levels were lower in the T2DM group than in the NC group (*P* < 0.001). Increased IRS-2 expression levels were observed in the SDC-L group (*P* < 0.001). The qRT-PCR and western blot results (Figures 5D,E) showed no differences in IRS-1 expression levels across the groups.

Glucose transporter proteins, including GLUT-2 and GLUT-4, were essential to regulate the liver and muscle glucose fluxes, respectively (28). In the present study, the GLUT-2 expression levels of liver tissue were lower in T2DM mice than in control mice (*P* < 0.001). The greatest increase in the GLUT-2 expression levels of liver tissue was observed in the SDC-H group (Figures 5B,C). The mRNA and protein expression levels of GLUT-4 were lower in the T2DM group than in the NC group (Figures 5D,E). In contrast, the GLUT-4 mRNA expression levels were increased in SDC intervention groups (*P* < 0.01). Moreover, the GLUT-4 protein expression levels were increased in the SDC-H group (*P* < 0.01).

### Effect of SDC on Colonic Microbiota

**Figure 6:**
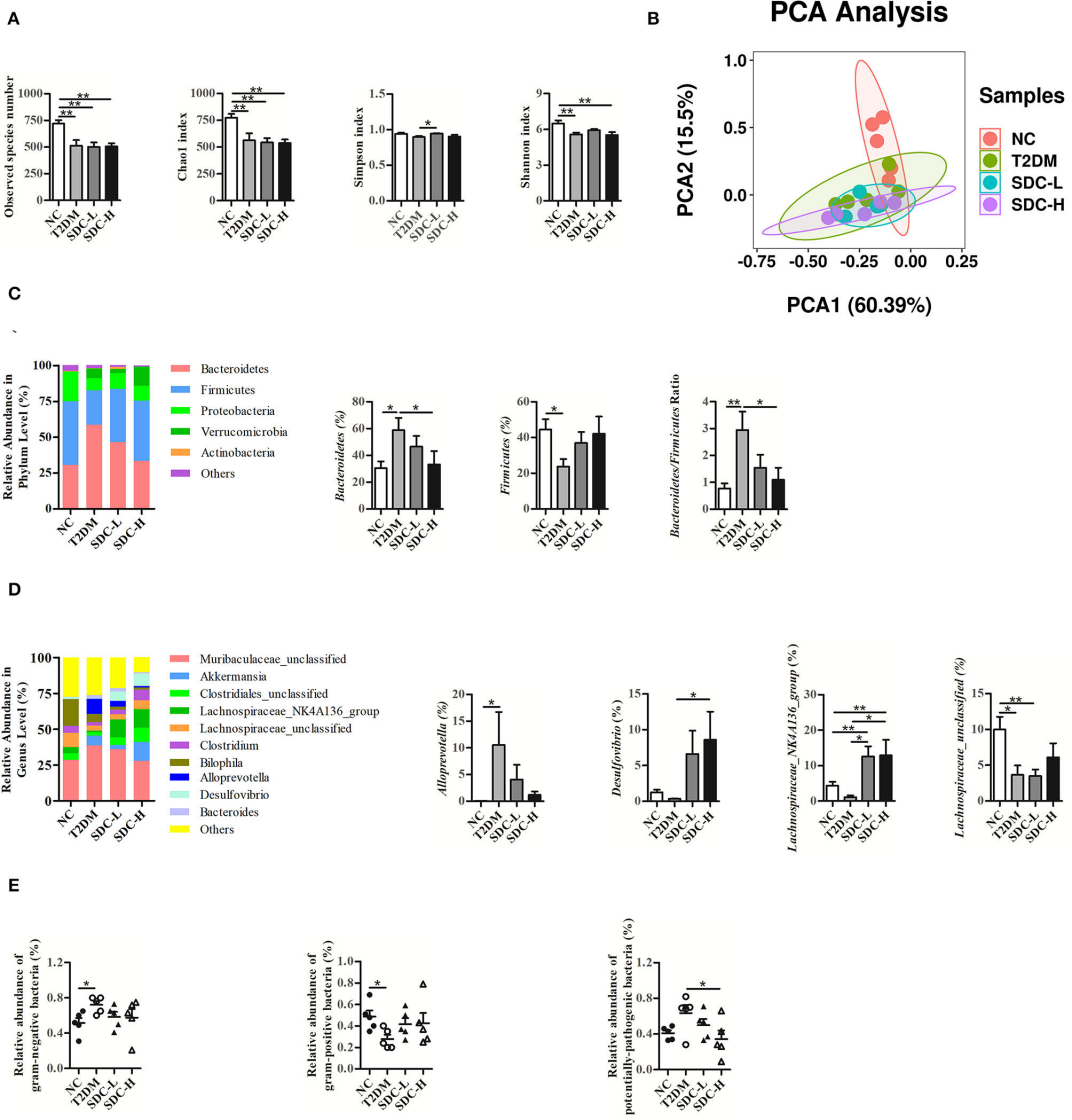
Alpha diversity **(A)** and beta diversity **(B)** of the mice; Change in phylum **(C)** and genus **(D)** with different treatments; The prediction of bacterial phenotypes in different groups **(E)**. *n* = 5, *P < 0.05, **P < 0.01.

As exhibited in Figure 6A, the alpha diversity of fecal flora in the T2DM group (vs. the NC group) was significantly decreased due to the effects of the combination of HFD and STZ, and SDC intervention only showed little effect on the alpha diversity of fecal flora (the Simpson index of the SDC-L group significantly increased compared to the T2DM group). Compared with the NC group, the T2DM group showed different beta diversity after the HFD and STZ injection (Figure 6B). However, SDC intervention did not significantly restore the beta diversity of intestinal flora.

As shown in Figure 6C, Bacteroidetes, Firmicutes, and Proteobacteria were the three dominant bacterial phyla of intestinal flora. The gut microbial composition of the T2DM group (vs. the NC group) was altered. The abundance of Bacteroidetes was increased, and that of Firmicutes was decreased in the T2DM group (vs. the NC group), leading to an increased Bacteroidetes/Firmicutes ratio. In contrast, the imbalanced Bacteroidetes/Firmicutes ratio was restored in the SDC-H group through the decrease in the abundance of Bacteroidetes and the increase in the abundance of Firmicutes.

At the genus level (Figure 6D), the relative abundances of *Muribaculaceae_unclassified, Akkermanisa, Alloprevotella* and *Bacteroides* in the T2DM group (vs. the NC group) were increased. The relative abundances of *Muribaculaceae_unclassified, Alloprevotella* and *Bacteroides* decreased after SDC intervention, while the relative abundance of *Akkermanisa* increased in the SDC-H group. In particular, the relative abundance of *Alloprevotella* was significantly increased (*P* < 0.05) in the T2DM group and reversed to some extent in SDC groups. In addition, the relative abundances of *Clostridiales_unclassified, Lachnospiraceae_NK4A136_group, Lachnospiraceae_unclassified, Clostridium, Bilophila*, and *Desulfovibrio* were decreased in the T2DM group (vs. the NC group). After SDC intervention, the relative abundances of these bacteria were reversed to some extent. Specifically, the relative abundance of *Desulfovibrio* was significantly increased in the SDC-H group (*P* < 0.05). Besides, SDC intervention significantly increased the relative abundance of *Lachnospiraceae_NK4A136_group* (*P* < 0.05).

Moreover, the prediction of bacterial phenotypes (Figure 6E) indicated that the relative abundance of gram-negative bacteria was significantly increased, and gram-positive bacteria was significantly decreased in the T2DM group (vs. the NC group). The SDC intervention showed no significant improvement of gram-negative bacteria and gram-positive bacteria. In addition, the relative abundance of potentially-pathogenic bacteria was significantly decreased in the SDC-H group (vs. T2DM group). The abundance changes of the above bacteria were altered by SDC, which might be related to its anti-diabetic effect.

## Discussion

Recently, growing evidence revealed that low glycaemic index (GI)/glycaemic load (GL) dietary patterns might be especially helpful for the management of diabetes (29). A slow digestion-oriented dietary approach might be beneficial for improving glucose homeostasis (30). SDC is typically found in low GI/GL foods and induces a low glycemic response (31). Previous studies showed that administration of SDC (isomaltodextrin, short-clustered maltodextrin, resistant starch, trehalose, and palatinose) might be beneficial for glycaemic control (32–36). The present study used cassava dextrin, maltodextrin, resistant starch, green banana powder, trehalose, and palatinose to create a high SDC content diet and studied its potential protective effect and mechanism against hyperglycemia and hyperlipidemia.

In the present study, SDC intervention lowered FBG, GHb levels, and AUC_0−120minGlu_, indicating that it improved glycemic control and glucose intolerance. Moreover, SDC elevated the pancreas index, inhibited insulin and glucagon release, decreased HOMA-IR, and increased QUICKI. These findings revealed that SDC ameliorated hyperglycemia and IR status in T2DM mice. In addition, SDC intervention inhibited TC and LDL-C levels, decreased the cell size in subcutaneous fat, and suppressed the lipid accumulation in the liver, subcutaneous fat and epididymal fat. These results suggested that SDC ameliorated hyperlipidemia status in T2DM mice. LEP and ADPN can improve insulin sensitivity in diabetic patients (16). In this study, SDC intake increased the levels of LEP and ADPN, indicating that it ameliorated IR through the insulin-sensitizing effect. Higher RES level is associated with reduced survival in T2DM patients, which might be due to the pro-inflammatory properties of RES (37). Herein, SDC administration decreased the RES level, suggesting that it inhibited the inflammation in T2DM mice.

The liver plays a crucial role in glucose homeostasis through balancing gluconeogenesis and glycogen synthesis (38). In this work, SDC intervention ameliorated the liver index increment and elevated the liver glycogen level, indicating that it might increase the glycogen synthesis and alleviate the IR status in the liver. IRS-1 and IRS-2, both insulin signaling elements, play different roles in insulin signaling, with IRS-1 required for insulin biosynthesis and secretion, while IRS-2 required the regulation of β-cell mass and proliferation (39). IRS-1 and IRS-2 show tissue-specific differences in mediating insulin action, with IRS-1 playing an essential role in skeletal muscle and IRS-2 in the liver (40). Therefore, the expression levels of IRS-1 and IRS-2 are detected in skeletal muscle and liver, respectively. The mRNA and protein expression analysis revealed that SDC improved the expression of IRS-2 in the liver but did not affect the expression of IRS-1 in skeletal muscle. These results suggested that SDC might relieve the IR status in T2DM mice *via* the regulation of β-cell mass and the proliferation by elevation of IRS-2 in the liver. GLUT-2 and GLUT-4 modulate the glucose uptake in the liver and skeletal muscle, respectively. The inactivation of GLUT-2 in the liver may lead to impaired glucose-stimulated insulin secretion (41). Impaired expression of GLUT-4 was linked to obesity, type 1 diabetes, and type 2 diabetes (42). In the present study, intervention with SDC increased liver GLUT-2 levels and skeletal muscle GLUT-4 levels in high-fat diet/streptozocin-induced T2DM mice, suggesting that SDC may regulate glucose metabolism through the regulation of GLUT-2 and GLUT-4.

Studies have reported that an imbalanced gut microbiota occurred in the onset and progression of T2DM, which makes the gut microbiota a good target (43, 44). A previous human study found that the Bacteroidetes/Firmicutes ratio was positively related to plasma glucose concentration (45). Moreover, a study found that intestinal Firmicutes levels increased in overweight men after consuming a diet high in resistant starch (46). An animal study revealed that polysaccharides from *Artocarpus heterophyllus* Lam. pulp (JFP-Ps) could modulate gut microbiota by increasing the abundance of Firmicutes and decreasing the abundance of Bacteroidetes (47). In the present study, SDC intervention increased the Firmicutes levels and reduced the elevated Bacteroidetes/Firmicutes ratio, indicating that SDC might improve the hyperglycemia status by maintaining gut microbiota balance. This result might be contrary to many previous studies (48). However, the Bacteroides/Firmicutes ratio that have been previously suggested as markers of metabolic disease did not show consistent associations with T2DM. The association between Bacteroides/Firmicutes ratio and diabetes pathogenesis remains controversial and unconfirmed (49). A recent study revealed that microbial co-occurrence could be an important factor to consider relating individual gut microbiome taxa to environmental factors and host health and disease (50). Thus, future studies might focus on the microbial co-occurrence instead of Bacteroides/Firmicutes ratio.

Regarding the genus levels, the Alloprevotella possessed the ability to produce short-chain fatty acids, and its abundance was reported to decrease in T2DM rats compared to the normal rats (51). Notably, the results of the present study were different from those of the study mentioned above, possibly due to the difference in the T2DM model used. The Desulfovibrio levels were reported to be increased in rats with a high-sugar diet or obese mice after treatment with theabrownin or blueberry polyphenols extract, respectively (52, 53). *Desulfovibrio vulgaris* from the *Desulfovibrio* genus has been shown to have a protective effect against hepatic steatosis (54). In the present study, the levels of the *Desulfovibrio* genus were increased, suggesting that SDC might improve the hyperlipidemia status by elevating the *Desulfovibrio* genus. The relative abundance of Lachnospiraceae NK4A136 was significantly increased following a Mediterranean diet, possibly due to improved insulin levels (55). Inulin supplementation might benefit health *via* elevation of the unidentified taxon belonging to a genus from the *Lachnospiraceae* family (56). Lotus seed resistant starch type 3 might promote the production of short-chain fatty acids through the increment of the *Lachnospiraceae* family (57). In the present study, the levels of *Lachnospiraceae_NK4A136_group* and *Lachnospiraceae_unclassified* were significantly increased after SDC intervention, suggesting that SDC intervention might be beneficial for T2DM through the elevation of the *Lachnospiraceae* family. Overall, high-fat diet/streptozocin induced aberrant gut microbiota, while SDC intervention can improve the hyperglycemia and hyperlipidemia status by inhibiting Bacteroidetes/Firmicutes ratio and elevating the *Desulfovibrio* and *Lachnospiraceae* genus levels.

As compared with SDC-L diet intervention, SDC-H diet intervention showed better protective effect against hyperglycemia, IR status and hyperlipidemia in T2DM mice. Besides, SDC-H diet showed significant protective effect on gut microbiota, while SDC-L diet showed little effect. However, as shown in the organ indices, SDC-H diet might induce negative effect on the kidney health. Thus, the SDC dose used in SDC-L and SDC-H groups might not be the optimum level to protect mice from T2DM. Future study need to find an appropriate dose of SDC to intervene T2DM. Since the pancreatic tissues and intestine tissues were not fixed in 10% neutral formalin solution immediately after they were excised, the present study lacked H&E staining and insulin immunofluorescence analysis of the pancreatic tissues, and the H&E staining analysis of intestinal histological changes. The pancreas atrophy, islet damage, and intestinal injury were not fully presented in this study, which might limit reflecting of the pancreas/intestine damage status in T2DM mice and connecting the gut microbial changes with metabolic and gut health. Thus, after studying an appropriate dose of SDC to intervene T2DM, future study may focus on the gut microbiota-bile acid-liver axis, tight junctions, FXR and TGR5 expression levels of intestine and liver tissues in depth to connect the gut microbial changes with metabolic and gut health. In addition, since STZ may be toxic to organs and tissues other than the pancreatic islet β-cells, HFD/STZ models could not precisely mimic the human condition. For this reason, this study may have limited clinical relevance and cannot extrapolate the findings directly to humans.

## Conclusion

The present findings demonstrated that SDC could control glycemia, improve IR status and decrease lipid accumulation in HFD/STZ induced diabetic mice. This effect may be due to the upregulation of IRS-2 and GLUT-2 in the liver, elevated GLUT-4 expression in skeletal muscle, and restored gut microbiota composition. All in all, SDC intervention can ameliorate hyperglycemia and hyperlipidemia in diabetic mice, providing a theoretical basis for the potential benefits of SDC in the management of T2DM.

## Data Availability Statement

The datasets presented in this study can be found in online repositories. The names of the repository/repositories and accession number(s) can be found below: NCBI (Accession: PRJNA799238).

## Ethics Statement

The animal study was reviewed and approved by Animal Ethical Committee of Soochow University.

## Author Contributions

Y-ZC, JG, Y-FD, LZ, and M-LL performed the experiments and analyzed the data. W-TC, H-QL, and YL designed the study and wrote, reviewed, and edited the manuscript. J-YX, H-TF, Y-HL, and L-QQ contributed to conceptualizing, drafting, and revising the manuscript. All authors contributed to the article and approved the submitted version.

## Funding

This work was funded by the China Central Government Guide the Development of Local Science and Technology Special Funds.

## Conflict of Interest

W-TC, H-QL, YL, and H-TF are employed by Inner Mongolia Dairy Technology Research Institute Co., Ltd. The remaining authors declare that the research was conducted in the absence of any commercial or financial relationships that could be construed as a potential conflict of interest.

## Publisher's Note

All claims expressed in this article are solely those of the authors and do not necessarily represent those of their affiliated organizations, or those of the publisher, the editors and the reviewers. Any product that may be evaluated in this article, or claim that may be made by its manufacturer, is not guaranteed or endorsed by the publisher.
